# Double anti-PL-7 and anti-MDA-5 positive Amyopathic Dermatomyositis with rapidly progressive interstitial lung disease in a Hispanic patient

**DOI:** 10.1186/s12890-020-01256-x

**Published:** 2020-08-15

**Authors:** Zi Ying Li, Evanpaul Gill, Fan Mo, Candice Reyes

**Affiliations:** 1 Department of Internal Medicine, University of California, San Francisco-Fresno, Fresno, CA USA; 2 UCSF Fresno Medical Education Program, Internal Medicine, 155 N Fresno St, Fresno, CA 93701 USA; 3 Division of Rheumatology, University of California–San Francisco, Fresno, CA USA

**Keywords:** Dermatomyositis, Myositis-specific autoantibodies, Anti-MDA5, Anti-PL-7, Anti-ARS, Rapid progressive interstitial lung disease

## Abstract

**Background:**

Each myositis-specific autoantibody (MSA) tends to have a distinct clinical presentation. Coexistence of MSAs do not commonly occur. If they do, it is unknown if there is an overlap of clinical features or prognostic implications. There are a few reported cases of overlap between these antibodies, mostly reported in patients with Japanese descent. Our aim for this case report is to turn more attention and interest for future MSA profile studies in the Hispanic population, which may hopefully spur better therapies if we realize the prognostic implications of certain myositis subsets including double-positive autoantibody syndromes.

**Case presentation:**

A 27-year-old Hispanic female was admitted to the medical intensive care unit due to acute hypoxemic respiratory failure secondary to acute respiratory distress syndrome (ARDS). She had failed conventional mechanical ventilation and was cannulated for venovenous extracorporeal membrane oxygenation (VV-ECMO) to manage her respiratory failure. She had erythematous scaly plaques on bilateral 3rd metacarpophalangeal joints on examination. Her autoimmune workup revealed positivity for both anti-PL-7(threonyl) and anti-melanoma differentiation-associated gene 5 (MDA5) autoantibodies. After extensive evaluation, it was concluded that she had rapidly progressive interstitial lung disease (RPILD) due to amyopathic dermatomyositis. Despite maximal medical management, she was ultimately transitioned to comfort care measures and expired.

**Conclusion:**

We would like to highlight the rarity of double antibody positive amyopathic dermatomyositis. This unique clinical presentation has only been reported in persons of Japanese descent. Our case is likely to be the first reported to occur in a person of Hispanic descent in the United States.

The rarity of our case could stimulate further study of overlapping MSA to understand its varied presentations and prognoses including possible tendency toward a rapidly progressive ILD phenotype. Earlier detection of these clinical syndromes can lead to better outcomes for patients with RPILD. This case report could also herald an increased recognition and understanding of MSA profile in the Hispanic population in the USA.

## Background

Myositis-specific autoantibodies (MSAs) are comprised of anti-aminoacyl-tRNA synthetase (anti-ARS) and non-anti-synthetase groups. These antibodies have differences in their pathophysiology profiles and clinical presentations, and are not usually known to coexist. Each anti-aminoacyl-tRNA synthetase (anti-ARS) antibodies tend to have its own unique presentation or combinations of myositis, interstitial lung disease (ILD) patterns, and skin manifestations. The MSAs include anti-EJ (glycyl) and anti-PL-7(threonyl) antibodies [1] Anti-melanoma differentiation-associated gene 5 (MDA5) antibody is one of the non-antisynthetase MSAs, which uniquely presents as clinically-amyopathic dermatomyositis (CADM), with its well-known potentially fatal course due to rapidly progressive ILD [2]. We only found two reported cases in Japan with double MSAs. The first one has anti-PL-7(threonyl) and anti-MDA5, and the second case with anti-EJ (glycyl) and anti-MDA5 [3, 4]. We report our case of a Hispanic patient instead of Asian descent as described in prior case reports, in addition to being possibly the first reported double-positive MSA-CADM case in the United States.

## Case presentation

A 27-year-old Hispanic female with chronic hirsutism and oligomenorrhea presented to an outside hospital in June 2019 with worsening chronic cough for 1 year. Per family, patient was in good health excepted for rash on her chest and knuckles that responded to topical hydrocortisone. There was no evaluation done in the outpatient setting for her chronic cough. She had presented to an urgent care where she was prescribed azithromycin 2 days prior to hospitalization.

**Fig. 1:**
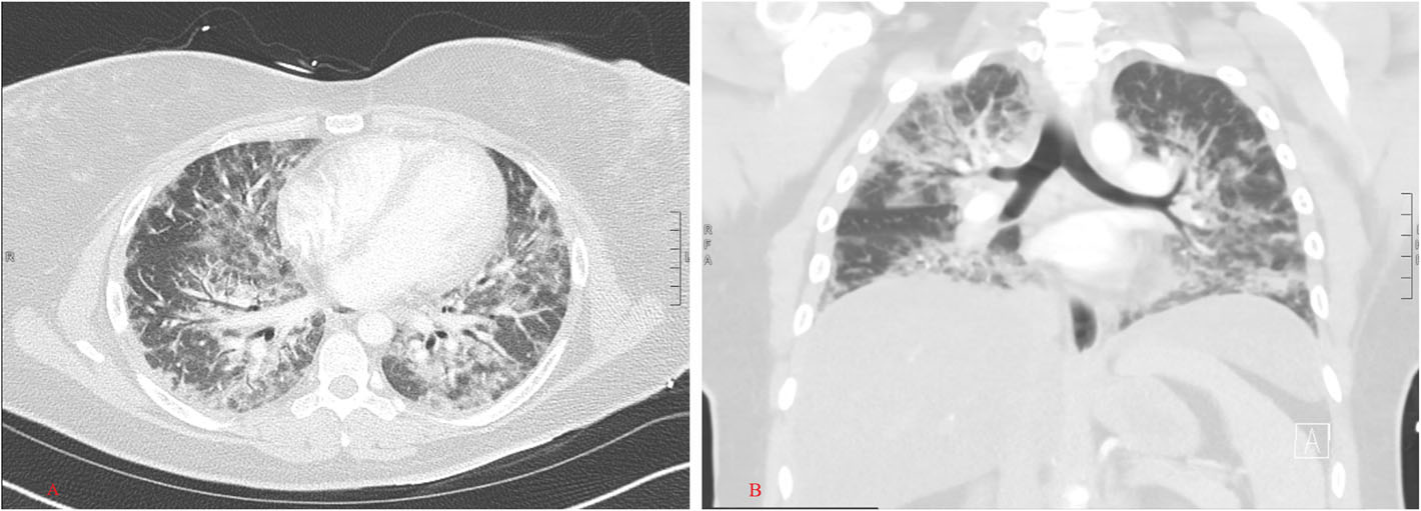
**a**-**b**: CT of the thorax showing diffuse ground glass opacities at the axial view (1a) and at the coronal view (1b)

On presentation to the outside hospital, she was febrile, tachycardic, tachypneic, with an oxygen saturation of 85% on nasal cannula at 15 L/min. Respiratory exam was significant for rhonchi bilaterally. Initial labs showed normal white blood cell count, normal creatine, and slightly elevated globulin with normal liver function tests. Her streptococcus pneumonia urinary antigen was positive. She was treated with a course of antibiotics. Computed tomography (CT) scan of the chest revealed no pulmonary embolus and demonstrated bilateral diffuse ground glass patchy opacities throughout the lungs (Fig. 1a-b).

She was initially admitted to the medical intensive care unit and intubated for hypoxemic respiratory failure due to acute respiratory distress syndrome (ARDS). Broad spectrum antibiotic therapy was started with intravenous piperacillin /tazobactam and levofloxacin. She was on maximal ventilatory support at the outside hospital and continued to remain hypoxemic with elevated peak and plateau pressures. Neuromuscular blockade was initiated, with only minimal improvement in oxygenation. Prone positioning was not attempted at the outside hospital. She was transferred to our hospital on day 7 of her course for evaluation of veno-venous extra corporeal membrane oxygenation (VV-ECMO).

On arrival to our hospital, her PaO2/FiO2 ratio was 40 despite maximal ventilator support and neuromuscular blockade. Given she had failed conventional ARDS treatments, VV ECMO was initiated. Bronchoscopy was performed and revealed no signs of diffuse alveolar hemorrhage (DAH), and infectious workup from bronchial lavage was negative.

Further workup at our center revealed mildly elevated aldolase, low-normal creatine kinase, and elevated transaminases. Autoimmune labs revealed positive antinuclear antibody (ANA), positive SS-A antibodies, elevated rheumatoid factor, elevated anti-PL-7 and anti MDA5 antibody levels detected by commercial immunoblot assay kit (Myositis Specific 11 Antibodies Panel, Quest Diagnostic, USA), and which was confirmed with repeated testing (Table 1).

**Table 1:** Laboratory test

Test name	Value (reference range)
**Hematology**	
White blood count	9.1 10^3^/uL at Day 1 of admission Range from 5.3–9.7 10^3^/uL during hospital stay (4.0–11.0 10^3^/uL)
Hemoglobin	12.8 g/dL at Day 1 of admission Range from 6.1–12.8 g/dL during hospital stay (12-16 g/dL)
Platelet count	260 10^3^/uL at Day 1 of admission Range from 68.0–260 10^3^/uL during stay (140–440 10^3^/uL)
**Chemistry**	
**Aldolase**	**12.4 (< 8.2 U/L**)
**Creatine kinase**	**17 to 26 U/L during admission (25–140 U/L)**
**Alanine aminotransferase (ALT)**	**15 to 227 U/L during admission (7-35 U/L)**
Lactic Acid	2.1 mmol/L at day 1 of admission Range from 0.9–4.1 mmol/L during admission stay
**Infectious**	
Culture, Blood	No growth
Culture, Respiratory aspiration	No growth
Culture, Fungal in respiratory aspiration	No growth
Culture, Urine	No growth
Cocci-Direct PCR	Negative
Legionella Urine antigen	Negative
Mycoplasma Pnuemoniae	Negative
Strep Pneumoniae Urine antigen	Positive
Upper respiratory viral panels	Negative
Influenza A/B PCR	Negative
HIV AB AG screen	Negative
**Autoimmune**	
Anti-neutrophil cytoplasmic antibody (ANCA)	Negative
Antinuclear antibody reflex (ANA)	Positive, titer 1:160
SS-A/Ro IgG antibody	1.2 positive (< 1.0 Negative)
SS-B/La IgG antibody	< 1.0Negative (< 1.0 Negative)
Anti-topoisomerase (SCL-70) IgG antibody	< 1.0 Negative (< 1.0 Negative)
Centromere auto antibody	< 1.0 Negative (< 1.0 Negative)
Double strand DNA (dsDNA)	1 IU/mL (< 5 IU/mL)
Smith IgG	< 1.0 negative (< 1.0 Negative)
Ribonucleic protein (U1 RNP/snRNP) IgG	< 1.0 negative (< 1.0 Negative)
Myositis panel (collection date: Hospital Day 8)	Jo-1 Ab < 11 (< 11 SI)
	**PL-7 Ab 60 (< 11 SI)**
	PL-12 Ab < 11 (< 11 SI)
	EJ Ab < 11 (< 11 SI)
	OJ Ab < 11 (< 11 SI)
	SRP Ab < 11 (< 11 SI
	Mi-2 Alpha Ab < 11(< 11 SI)
	Mi-2 Beta Ab < 11 (< 11 SI)
	**MDA-5 Ab 89 (< 11 SI)**
	TIF-1y Ab < 11 (< 11 SI)
	NXP-2 Ab < 11 (< 11 SI)
Myositis panel (collection date: Hospital Day 16)	Jo-1 Ab < 11 (< 11 SI)
	**PL-7 Ab61 (< 11 SI)**
	PL-12 Ab < 11 (< 11 SI)
	EJ Ab < 11 (< 11 SI)
	OJ Ab < 11 (< 11 SI)
	SRP Ab < 11 (< 11 SI
	Mi-2 Alpha Ab < 11(< 11 SI)
	Mi-2 Beta Ab < 11 (< 11 SI)
	**MDA-5 Ab 78 (< 11 SI)**
	TIF-1y Ab < 11 (< 11 SI)
	NXP-2 Ab < 11 (< 11 SI)

**Fig. 2:**
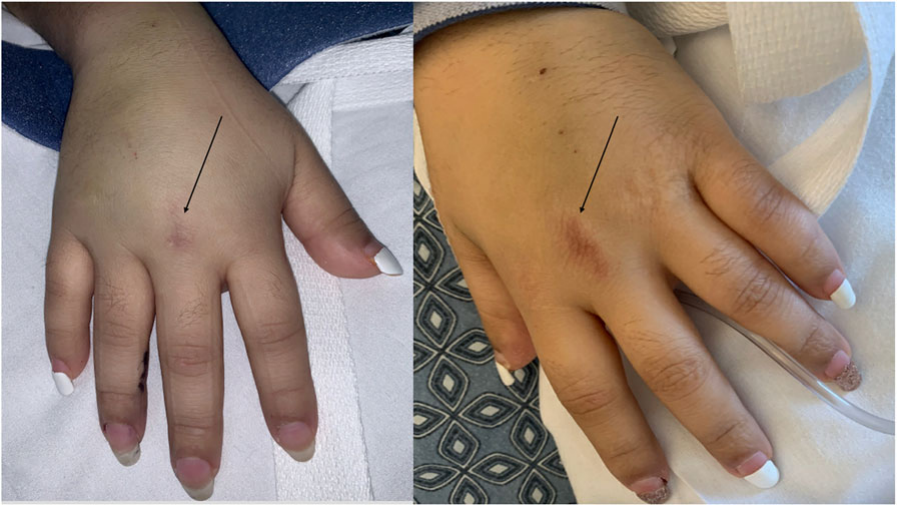
Bilateral *forme fruste* of Gottron’s patches at the third metacarpophalangeal joints (black arrows)

Jo-1 (antihistidyl-tRNA synthetase), PL-7 (threonyl), PL-12 (alanyl), EJ (glycyl), OJ (isoleucyl), Mi-2 (Mi-2/nucleosome remodelling and deacetylase (NuRD), SRP (signal recognition particle), MDA5 (melanoma-differentiation associated gene 5), TIF-1 y (intermediary factor 1-gamma), NXP-2(nuclear matrix protein),

Physical examination revealed diffuse facial hair and hyperpigmented patches at the dorsum of bilateral third metacarpophalangeal joints (Fig. 2). These skin features, along with the lack of muscle weakness and unremarkable muscle enzymes, CT chest findings of bilateral ground glass opacities, double positive MSAs (anti-PL-7 and MDA5), and negative infectious work up, all led to a diagnosis of acute hypoxemic respiratory failure secondary to rapidly progressive interstitial lung disease (RPILD) due to CADM.

Patient remained on maximal support from the ECMO circuit and the ventilator. Pulse doses of methylprednisolone 1 g daily was initiated on day 10, intravenous immune globulin (IVIG) was given on day 17, and rituximab was given on day 19. She was then transitioned to methylprednisolone 60 mg daily on day 20. Repeat CT chest on day 21 showed worsening of consolidation and persistent patchy GGO bilaterally.

She was eventually transferred to a lung transplant center on day 24 given no significant improvement despite on VV-ECMO and maximal ventilator support for 17 days. After a detailed evaluation by multi-disciplinary team at the lung transplant center, she was deemed not to be a lung transplant candidate. Her family had decided to transition to comfort care on day 33 and patient expired.

## Discussion and conclusions

The clinical features associated with certain MSA can usually be distinct from one another. Anti PL-7 antibodies are classified under the anti-ARS group. These antibodies are associated with a median age of clinical onset of 60 years old with 90% having non-specific interstitial pneumonia (NSIP) on HRCT and elevated serum creatinine kinase [5]. Anti-MDA5 antibody cases were mostly reported in Japan and rural China; 90% of them have dermatomyositis related skin findings and pulmonary findings of acute ILD with consolidation and ground glass attenuation [5]. Chest CT of MDA5-ILD is reportedly characterized by lower lung consolidation or a random GGO pattern with absence of intralobular reticular opacities and traction bronchiectasis [6]. Anti-MDA5 associated ILD carry a poorer prognosis as compared to anti-ARS associated ILD [7].

In the case reported by Naniwa et al, they described a patient who tested positive for PL-7 and MDA5 antibodies and had chest CT features of the corresponding antibodies at the time of onset for RPILD, which had improved pulmonary function after high dose of IVIG [3]. In another case written by Takeuchi et al*,* their patient’s double MSAs dermatomyositis-ILD was associated with anti-EJ and anti-MDA5 antibodies. During the initial course, chest CT had peripheral ground glass attenuation, marked reticulation, traction bronchiectasis and volume loss in the lower lung field bilaterally that were consistent with anti-ARS ILD associated disease, even though patient had anti-EJ and anti-MDA5 positive. The patient later gained anti-MDA5 ILD associated features during an acute exacerbation phase, at which time only anti-MDA5 antibodies were positive [4].

In contrast to these previous two cases of anti-ARS antibodies with co-existent anti-MDA5, our patient’s chest CT had extensive ground glass opacities bilaterally without bronchiectasis, which is more indicative of the pulmonary features for anti MDA5 ILD. Interestingly, she was found to have positive testing for both anti-PL7 and anti-MDA5 on repeated MSAs panel testing. Further comparison among these three cases is listed at Table 2.

**Table 2:** Comparison on coexistent anti-aminoacyl-tRNA synthetase (anti-ARS)-interstitial lung disease (ILD) dermatomyositis with anti-melanoma differentiation-associated gene 5 (MDA5)-ILD dermatomyositis

	Naniwa et al. [3]	Takecuchi et al. [4]	Current case
Patient ethnicity	Japanese	Japanese	Hispanic
Gender	Male	Female	Female
Age	70-year-old	53-year-old	27-year-old
Anti-ARS antibodies	Yes, anti-threonyl-transfer RNA synthetase (anti-PL-7)	Yes, Anti-glycyl-tRNA synthetase (anti-EJ)	Yes, anti-threonyl-transfer RNA synthetase (anti-PL-7)
Anti-MDA-5 antibodies	Yes	Yes	Yes
Diagnostic testing tool	Immunoprecipitation assay	Immunoprecipitation assay	Commercial Immunoblot assay
Skin manifestation	Facial rash, a V-sign rash, a **periungual erythema**, and nail fold bleeding	heliotrope rash, facial erythema, **Gottron papules** with some shallow ulcers, mechanic’s hands and **periungual erythema**	*forme fruste* of **Gottron’s patches**
Pulmonary Manifestation	Fine crackles were heard bilaterally in the lower lung field	Lung auscultation identified considerable fine crackles bilaterally	Diffuse crackles at lung auscultation bilaterally
Chest computed tomography findings	Not done	**Initial presentation with anti-EJ only:** lower peripheral reticulation and ground-glass attenuation (GGA) **15 years after onset during exacerbation with anti-MDA5 only:** rapidly progressive course with newly developed random GGA	Extensive ground glass opacities bilaterally without bronchiectasis

Different from the previous two cases which used immunoprecipitation methods, our case was tested with immunoblot assay MSA panel testing via Quest Diagnostics. This was repeated later to ensure no false positivity, and results remained positive at similar levels for each antibody. According to the study done by Cavazzana *et.al,* there was lower agreement rate for anti-Jo antibodies, and higher agreement rate for anti-MDA-5 antibodies when analyzing the performance of immunoblot assay and immunoprecipitation, but there has been no comparison between these 2 methods for Anti-PL-7 antibodies [8].

Dermatomyositis with unique MSA profiles are increasingly recognized for its varied presentations but with unclear overlapping prognoses, and even more elusive associations with malignancies. Certain features, such as RPILD may be a common denominator for some of the MSA profiles. Of note, the hyperpigmented patches over bilateral third MCP joints are likely a *forme fruste* of Gottron’s patches. Cognizance of these rashes in its potentially slightest expression may give a clue to solidify a dermatomyositis diagnosis.

Survival rates vary, with anti-MDA5 having the poorest reported survival [7]. Since most of the anti-MDA5 associated dermatomyositis cases were mostly reported in Asian countries, it is still unclear if the prognosis will be different in other ethnic groups, or if it will be different for similar ethnic groups living outside the Asian geographic regions.

The pathogenesis of double positive MSAs in amyopathic dermatomyositis is largely unclear given the rarity of reported data. There is neither MSA-specific therapy guideline for CADM/ILD, nor for double-antibody positive cases. Glucocorticoid monotherapy or in combination with immunosuppressants, IVIG, cyclophosphamide, rituximab, plasmapheresis have been observed in current practices with varied outcomes.

Further study into MSA profiles in the Hispanic population will be helpful to understand the prevalence, prognoses, and outcomes of these potentially life-threatening myositis syndromes. Such studies may lead to further insight into the pathogenesis of myositis and whether differences in profile patterns translate into varying clinically outcomes.

## Data Availability

Not applicable.

## Abbreviations

ARDS: Acute respiratory distress syndrome
CT: Computed tomography
GGO: Diffuse ground glass opacities
HRCT: High resolution computed tomography
IVIG: Intravenous immunoglobulin
MSA: Myositis-specific autoantibody
Anti-ARS: Anti-aminoacyl-tRNA synthetase
RPILD: Rapid progressive interstitial lung disease
VV-ECMO: Veno-venous Extra Corporeal Membrane Oxygenation
